# Therapeutic targets of formononetin for treating prostate cancer at the single-cell level

**DOI:** 10.18632/aging.205935

**Published:** 2024-06-13

**Authors:** Jiawei Li, Zhaoquan Huang, Ping Wang, Rong Li, Li Gao, Keng Po Lai

**Affiliations:** 1 Department of Urology Surgery, The Second Affiliated Hospital of Guilin Medical University, Guilin Medical University, Guilin, PR China; 2 Key Laboratory of Environmental Pollution and Integrative Omics, Guilin Medical University, Education Department of Guangxi Zhuang Autonomous Region, Guilin Medical University, Guilin, PR China; 3 Guilin Medical University, Guilin, PR China

**Keywords:** network pharmacology, formononetin, prostate cancer, single cell, therapeutic targets, immune response, heterogeneity

## Abstract

Prostate cancer is one of the serious health problems of older male, about 13% of male was affected by prostate cancer. Prostate cancer is highly heterogeneity disease with complex molecular and genetic alterations. So, targeting the gene candidates in prostate cancer in single-cell level can be a promising approach for treating prostate cancer. In the present study, we analyzed the single cell sequencing data obtained from 2 previous reports to determine the differential gene expression of prostate cancer in single-cell level. By using the network pharmacology analysis, we identified the therapeutic targets of formononetin in immune cells and tissue cells of prostate cancer. We then applied molecular docking to determine the possible direct binding of formononetin to its target proteins. Our result identified a cluster of differential gene expression in prostate cancer which can serve as novel biomarkers such as immunoglobulin kappa C for prostate cancer prognosis. The result of network pharmacology delineated the roles of formononetin’s targets such CD74 and THBS1 in immune cells’ function of prostate cancer. Also, formononetin targeted insulin receptor and zinc-alpha-2-glycoprotein which play important roles in metabolisms of tissue cells of prostate cancer. The result of molecular docking suggested the direct binding of formononetin to its target proteins including INSR, TNF, and CXCR4. Finally, we validated our findings by using formononetin-treated human prostate cancer cell DU145. For the first time, our result suggested the use of formononetin for treating prostate cancer through targeting different cell types in a single-cell level.

## INTRODUCTION

Prostate cancer ranks as the second most common cancer in men after lung cancer, posing a serious threat to the life and health of middle-aged and elderly men [1]. And, it is one of the leading causes of cancer-related deaths in men. Early symptoms of prostate cancer are not obvious, and most early diagnoses of prostate cancer are detected by prostate-specific antigen (PSA) screening and magnetic resonance imaging (MRI) [2]. The symptoms present in each patient are variable, due to the heterogeneity of prostate cancer, in which, the size of the tumor, the degree of malignancy, and metastatic ability are different [3]. Also, the high tumour heterogeneity in prostate cancer was a challenge for clinical disease management and a serious problem for molecular stratification of patients [4]. Single-cell RNA sequencing (scRNA-seq) method can analyze tumor tissue heterogeneity on a large scale and at the level of individual cells, allowing for more precise detection of changes during tumor development [5]. Furthermore, scRNA-seq can show the differential gene expression in individual cells which help to understand the detail process of disease development [6]. The stage of prostate cancer is determined using Gleason score, the higher the Gleason score, the more malignant the prostate cancer is, the more likely it is to progress and metastasize [7]. The main options for the treatment of prostate cancer are surgery, chemotherapy, and radiotherapy [8]. There is an urgent need to discover new prostate cancer treatment methods.

Estrogen receptors (ERs) include ERα and ERβ expressed predominantly in prostate stromal cells and prostate epithelial cells, respectively [9]. They were reported to play important roles in prostate cancer tumorigenicity. For instance, the activation of ERα and ERβ promoted the migration, cell invasion and colony formation abilities in hormone-independent prostate cancer cells [10]. In addition, the development of prostate cancer into denuded resistant prostate cancer is mediated by ERα and ERβ [11]. So, targeting ER could be an option for discovery of novel compound for treating prostate cancer. Formononetin, a natural isoflavone phytoestrogen, was mainly extracted from the Chinese herbs *Astragalus membranaceus* and Red Clover [12]. Formononetin exhibits estrogen-like effects through its interaction with ER, especially ERβ [13]. It has been reported that formononetin could be a promising anticancer drug. For instance, formononetin can inhibit cancer cell growth, promote cancer cell apoptosis and activate tumor suppressor genes to inhibit cancer development and progression [14]. It has been shown the anti-tumoral effects of formononetin on bladder cancer [15], prostate cancer [16], ovarian cancer [17], and non-small cell lung cancer [18]. However, the detail molecular mechanisms underlying the anti-prostate cancer roles of formononetin, especially in single-cell level, is still largely unknown.

In the present study, we analyzed the singe-cell sequencing data of prostate cancer obtained from 2 previous reports to determine the differential gene expression in single-cell level [19, 20]. Then we applied network pharmacology followed by systematic bioinformatics analysis including Gene Ontology (GO), Kyoto Encyclopedia of Genes and Genomes (KEGG), and molecular docking analysis to investigate the targets and molecular mechanisms of formononetin for treating prostate cancer. Our results showed that formononetin could target genes associated with the immune responses and metabolisms. Our data, for the first time, suggested that formononetin could be a novel compound for treating prostate cancer through targeting immune cell and tissue cell clusters. The results of this study provide ground information of formononetin against prostate cancer in single-cell level.

## MATERIALS AND METHODS

### Single cell sequencing data download and analysis

By searching with the keywords “prostate cancer” and “single cell”, the gene expression datasets GSE193337 and GSE153892 of prostate cancer single cell data were downloaded from the GEO database (https://www.ncbi.nlm.nih.gov/geo/). 4 sets of prostate cancer tissue and normal tissue were extracted from the GSE193337 data [19], and 3 sets of prostate cancer tissue and normal tissue were extracted from the GSE153892 data [20]. Single cell data were analyzed using R packages including “Seurat”, “cowplot”, “BiocManager”, “SingleR”, “dplyr”, “tidyverse”, and “patchwork”. The data were merged into a catalogue vector and a Seurat analysis object was created with the filtering criteria of fewer than genes expressed in 3 cells and fewer than 200 genes expressed in cells, the PercentageFeatureSet function was used to calculate the percentage of mitochondria-related genes in the data, and the data were quality controlled with the criteria of more than 200 genes per cell, less than 3,000 genes per cell, and less than 20% mitochondria, the cells that failed to pass the criteria were eliminated [19]. The data were normalized to extract the 2000 genes with high coefficients of variation between cells, and the data were merged with canonical correlation analysis anchors to eliminate the batch effect between two different sets of data from different sources. PCA principal component analysis was performed on the data, and the cells were classified into different clusters by Umap cluster analysis. Cell type annotation for each cluster was completed by SingleR automated annotation package [21]. The data were grouped according to their origin, with those derived from normal tissues named “N” and those derived from cancerous tissues named “T”, and then “N” and “T” were combined with different cell types separately. The FindMarkers function was used to obtain the differential gene expression between the same cell types from cancerous tissue and normal tissue, and the gene with adjusted *p*-value < 0.05 were considered as differentially expressed genes (DEGs).

### Identification of formononetin’s targets to prostate cancer in single-cell level

The Swiss Target Prediction database [22], PharmMapper database [23] and SuperPred database [24] were used to obtain the formononetin associated genes, which were corrected by the UniprotKB database [25]. The genes were overlapped with the DEGs of the prostate cancer to determine the targets of formononetin for treating prostate cancer in single-cell level.

### Gene Ontology (GO) and Kyoto Encyclopedia of Genes and Genomes (KEGG) pathway enrichment analysis

The targets were imported into the “ClusterProfiler” package [26] and “GOplot” package [27] in R language for GO and KEGG enrichment analysis and visualization, the processes and pathways with *p*-value < 0.05 were considered as statistically significant. The results were presented as bubble plots and Circos plot.

### Molecular docking

The chemical structure of formononetin was obtained from PubChem database [28]. The protein structures of CD74, INSR, TNF, and CXCR4 were obtained from Protein Data Bank (PDB) database [29]. The corresponding proteins were processed using AutoDock Tools 1.5.6, of the AutoDock Vina software [30], to add hydrogen, Gasteiger charges, and merge non-polar hydrogens from the original pdb file format to the pdbqt file format recognized by the AutoDock program, to provide a ligand basis for subsequent docking. The reasonableness of the docking parameter settings was judged based on the magnitude of the root mean square deviation (RMSD) of the docked ligand molecules from the original ligand molecules. RMSD ≤ 4 Å was used as the threshold value for the conformational match of the docked ligand with the original ligand.

### Prostate cancer cell culture and treatment

Human prostate cancer cell DU145 (Wuhan Pricella Biotechnology Co., Ltd., China) was cultured in Eagle’s Minimum Essential Medium supplemented (Solarbio, China) with 10% fetal bovine serum and 1% Penicillin/Streptomycin/Amphotericin B, sterile solution (Solarbio) at 37°C in 5% CO_2_. DU145 cells were seeded into 6-well plates at 3 × 10^5^ cells per well, and were treated with 0 and 100 μM of formononetin (Shanghai Yuanye Bio-Technology Co., Ltd., China) for 48 hrs. Then the cells were harvested for quantitative-PCR (qPCR) analysis and Western blotting (WB).

### Quantitative PCR (qPCR) analysis

Total RNA was isolated from the cells using the RNA simple Total RNA Kit (Tiangen, China) and was reverse transcribed according to the manufacturer’s protocol. Gene expression was quantified using MonAmpTMSYBR Green qPCR Mix (Low ROX, Monad) and expression level was calculated using the 2^−ΔΔCT^ method. GAPDH was used as internal reference for normalization.

### Western blotting

Protein was extracted from the cells using RIPA lysis buffer (Solarbio) and was quantified by using the BCA protein assay kit (Beyotime, China). 20 μg of protein was separated by using SDS PAGE and electro-transferred onto PVDF membranes (Merck, Germany). The membrane was then incubated with specific primary antibodies CD74 (Bioss, USA) and TNF (Bioss) overnight at 4°C. The membrane was washed with TBST thrice for 10 min. After washing, the membrane was incubated with HRP anti-coupling secondary antibody for 1 hour. The membrane was washed with TBST thrice for 10 min. Finally, the expression of protein was assessed by exposing to ECL kit (Affinity, USA), and the gel imaging system (Invitrogen, USA).

## RESULTS

### Differential gene expression in prostate cancer in single-cell level

**Figure 1:**
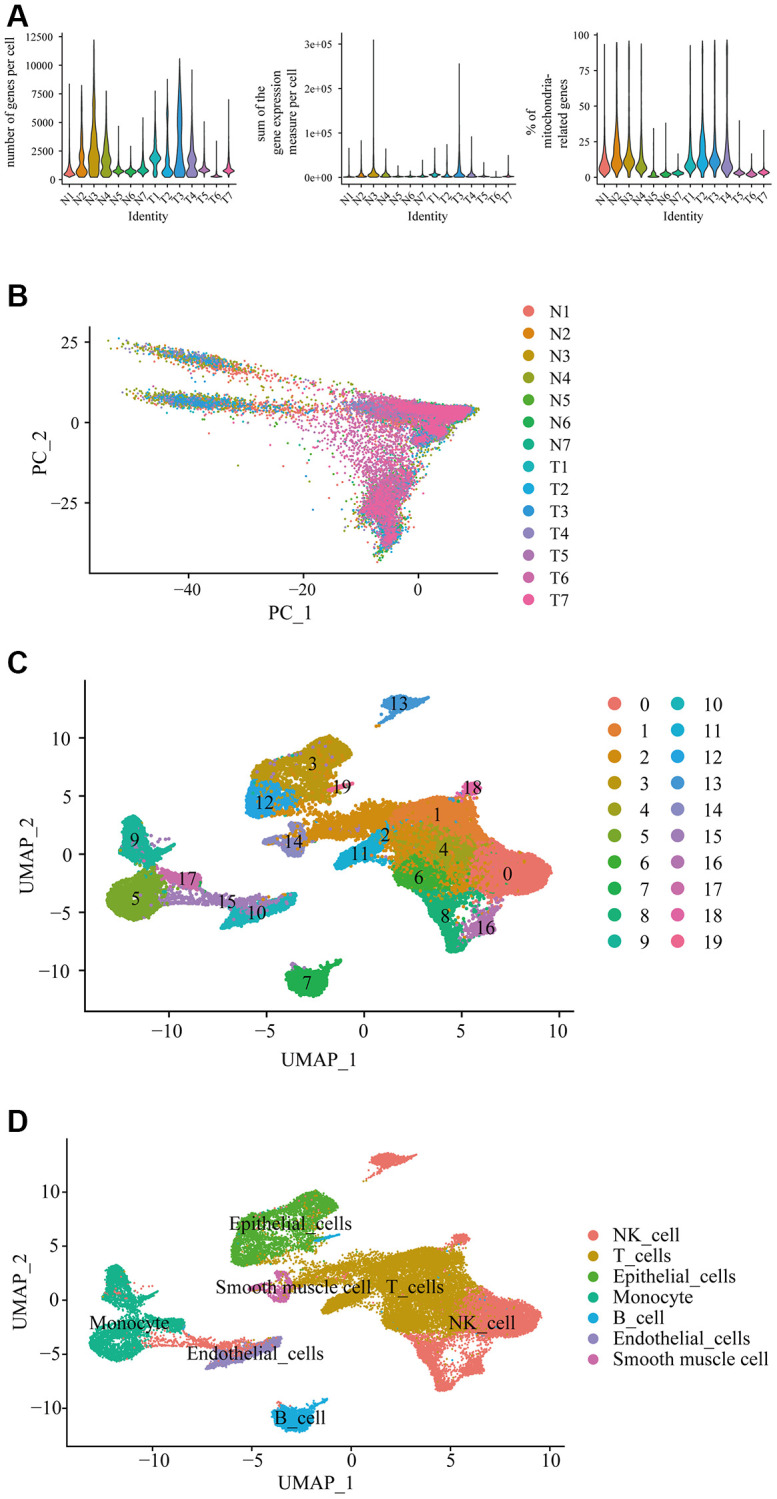
**Differential gene expression in prostate cancer in single-cell level.** (**A**) Single cell sequencing analysis using the downloaded dataset showed the number of genes measured per cell, the sum of the gene expression measured per cell, and the percentage of mitochondria-related genes in the prostate cancer in single-cell level. N represented adjacent normal tissues; T represented prostate cancerous tissues. (**B**) Principal component analysis (PCA) showed the similarity of the samples. (**C**) Uniform manifold approximation and projection (Umap) classified the single cell into 19 cell clusters. (**D**) Each cluster was classified into different cell type using SingleR automatic annotation package, including B cell, NK cell, T cell, monocyte, epithelial cells, endothelial cells, and smooth muscle cells.

When we combined and analyzed the 2 datasets of prostate cancer single cell data, we identified the number of genes measured per cell, the sum of the gene expression measured per cell and the percentage of mitochondria-related genes (Figure 1A). The distribution of each cell in different samples was shown in the PCA plot, and it can be found that there is no significant batch effect among the 14 samples under canonical correlation analysis (Figure 1B). In the uniform manifold approximation and projection (Umap) cluster analysis, 19 cell clusters were identified (Figure 1C). Each cluster was attributed to different cell type using SingleR automatic annotation package, in which clusters 0, 8, 13, 15, 16, and 18 belong to natural killer cell; clusters 1, 2, 4, 6, and 11 belong to T cell; clusters 7 and 19 belong to B cell; clusters 5, 9, 17, belong to monocyte; clusters 3 and 12 belong to epithelial cell; cluster 10 belongs to endothelial cells; and cluster 14 belongs to smooth muscle cells (Figure 1D). It should be noted that the automated annotation package annotated the clustering 14 as chondrocytes, in which, it should not be in the prostate tissue. So, we redid the analysis manually to analyze the top 10 expressed genes in the cluster 14, including TAGLN, RGS5, IGFBP5, ACTA2, IGFBP7, CALD1, MYL9, TPM2, TIMP3, NR2F2 through the online website Cell Taxonomy (https://ngdc.cncb.ac.cn/celltaxonomy/), we found that TAGLN, MYL9, and TPM2 genes indeed highly expressed in smooth muscle cell. Then we compared the gene expression profile between cancer tissues (named “T”) and the adjacent normal tissues (named “N”) to determine the DEGs in prostate cancer in single-cell level, in which there were 28 DEGs in B cell (Table 1); 31 DEGs in NK cell (Table 2); 31 DEGs in T cell (Table 3); 44 DEGs in monocyte (Table 4); 257 DEGs in epithelial cells (Supplementary Table 1); 30 DEGs in endothelial cells (Table 5); and 37 DEGs in smooth muscle cells (Table 6).

**Table 1:** DEGs in B cell.

**Gene symbol**	**log2 fold change (cancer/normal)**	**adjusted *p*-value**
KLF2	−0.39	2.57E-08
HLA-DRB5	−0.31	2.70E-12
NR4A1	−0.31	0.0045
HLA-DRA	−0.30	2.23E-11
CD74	−0.29	9.07E-12
CD79A	−0.29	1.49E-07
HLA-DQA2	−0.29	1.54E-10
RALGPS2	−0.28	4.08E-07
MEF2C	−0.28	0.0033
HLA-DQB1	−0.28	5.23E-06
XCL2	0.25	1.62E-23
DUSP4	0.25	0.037
CTSW	0.29	2.29E-16
CCL20	0.30	0.0044
IGHGP	0.32	0.00056
ANKRD28	0.32	1.62E-10
GZMA	0.32	2.17E-21
IFNG	0.35	0.0011
XBP1	0.35	5.19E-15
XCL1	0.37	8.20E-14
PRDM1	0.38	1.55E-08
IGHA2	0.39	2.73E-09
GNLY	0.43	3.13E-08
ITM2A	0.43	0.0038
CD7	0.50	1.01E-08
CYTOR	0.53	3.16E-05
CXCL8	0.67	0.015
TNFAIP3	0.68	5.58E-12

**Table 2:** DEGs in NK cell.

**Gene symbol**	**log2 fold change (cancer/normal)**	**adjusted *p*-value**
CCL4	−0.30	1.59E-08
CXCR4	−0.27	0.017
HSPA1A	−0.27	5.30E-06
NKG7	−0.25	6.97E-24
BAG3	0.25	5.11E-39
CTSS	0.26	0.0021
HLA-DRB5	0.27	0.031
FCGRT	0.28	1.41E-40
INSR	0.31	9.56E-31
ATF3	0.31	5.83E-15
OLR1	0.31	1.56E-07
IGKC	0.35	3.24E-16
HLA-DMB	0.35	3.87E-05
CD14	0.36	5.10E-23
CD83	0.36	0.00032
PHACTR1	0.37	4.22E-08
MARCKS	0.39	1.91E-41
IER3	0.40	1.08E-07
LYZ	0.41	9.52E-06
CTSB	0.44	5.44E-13
C15orf48	0.47	1.44E-27
AIF1	0.47	5.96E-06
CST3	0.48	9.02E-27
CCL20	0.51	5.30E-51
C1QB	0.51	1.10E-05
CXCL8	0.53	2.29E-09
C1QC	0.56	1.33E-13
MS4A6A	0.58	2.21E-31
IL1B	0.60	4.80E-21
G0S2	0.61	1.28E-10
CD74	0.61	4.78E-11

**Table 3:** DEGs in T cell.

**Gene symbol**	**log2 fold change (cancer/normal)**	**adjusted *p*-value**
CCL4	−0.59	9.15E-15
HBA2	−0.59	2.93E-29
TNF	−0.49	6.88E-10
CCL5	−0.48	2.79E-47
HSPA1B	−0.41	2.50E-09
IGFBP7	−0.41	3.79E-67
RGS5	−0.39	1.63E-40
CALD1	−0.38	1.83E-84
HSP90AA1	−0.37	6.19E-26
DNAJB1	−0.34	1.78E-12
TAGLN	−0.34	2.49E-29
GZMK	−0.28	2.30E-11
XCL2	−0.28	2.53E-29
JUN	−0.27	1.12E-21
SCGB1A1	−0.27	5.61E-32
FOS	−0.26	4.79E-14
XCL1	−0.26	1.26E-47
PLCG2	−0.26	8.57E-58
PLIN2	0.25	5.25E-11
PMAIP1	0.26	1.70E-27
ZFAND2A	0.26	0.0010
GNLY	0.26	3.07E-26
FABP5	0.26	0.018
PLAUR	0.27	9.52E-08
AC020916.1	0.27	2.68E-07
IL1B	0.30	2.88E-26
HLA-DRB1	0.31	5.49E-10
G0S2	0.34	0.021
GZMB	0.37	4.76E-36
B4GALT1	0.38	6.12E-10
CXCL8	0.46	9.82E-35

**Table 4:** DEGs in monocyte.

**Gene symbol**	**log2 fold change (cancer/normal)**	**adjusted *p*-value**
HBB	−1.40	1.19E-05
HBA2	−1.09	5.07E-49
HLA-DRB5	−0.41	0.00039
CD14	−0.41	1.08E-11
LYVE1	−0.40	4.15E-07
CEBPD	−0.39	6.45E-07
FRMD4A	−0.36	0.0010
S100A8	−0.34	0.00124
PLCG2	−0.33	7.55E-09
THBS1	−0.32	0.00021
VCAN	−0.32	2.84E-09
EPB41L2	−0.31	0.00010
CYBB	−0.30	3.35E-05
AQP9	−0.30	0.020
HLA-DRA	−0.29	3.63E-08
DST	−0.29	7.68E-06
C1QB	−0.29	0.0044
NRP1	−0.28	0.0021
HLA-DPA1	−0.28	4.23E-05
SAT1	−0.27	3.29E-07
CCL8	−0.27	0.00060
C1QC	−0.26	0.050
FCN1	−0.26	4.12E-07
CD74	−0.26	2.81E-07
TRAC	0.26	0.0010
DUSP4	0.28	5.05E-06
GPNMB	0.28	0.0019
ANKRD28	0.30	0.047
GEM	0.31	0.0027
CCR7	0.34	8.48E-05
MT1H	0.35	1.26E-24
CCL5	0.36	7.74E-05
PRDM1	0.37	0.022
TRBC1	0.37	0.00022
KLRB1	0.37	8.11E-13
IL32	0.38	4.51E-07
ATF3	0.38	0.013
PIM2	0.39	3.08E-10
SDS	0.41	1.94E-12
MT1F	0.48	0.014
GZMB	0.50	4.01E-15
CXCL10	0.53	7.68E-15
FABP5	0.55	3.04E-06
MT1E	0.77	0.022

**Table 5:** DEGs in endothelial cell.

**Gene symbol**	**log2 fold change (cancer/normal)**	**adjusted *p*-value**
HBB	−0.91	3.25E-15
CXCL9	−0.89	2.85E-11
HBA2	−0.83	1.55E-36
HBA1	−0.41	9.42E-16
ESM1	−0.40	0.00023
DSP	−0.39	3.79E-15
PDK4	−0.38	0.00012
PIGR	−0.36	4.92E-67
WFDC2	−0.34	1.39E-55
FAM13C	−0.31	9.99E-06
KRT19	−0.31	4.04E-40
LEF1	−0.30	0.0034
TGFB2	−0.30	4.42E-06
SCGB1A1	−0.30	1.69E-42
TSHZ2	−0.29	0.019
FN1	−0.29	0.033
KCNQ1OT1	−0.28	0.0042
NRN1	−0.28	0.00058
DNASE1L3	−0.28	0.0016
RARRES1	−0.28	9.01E-38
MALL	−0.27	0.011
GPIHBP1	−0.27	3.01E-12
FOLH1	0.27	0.021
TPSAB1	0.29	2.19E-12
C1QA	0.33	1.98E-07
C15orf48	0.35	4.49E-26
SPP1	0.38	9.47E-05
IL1B	0.39	1.03E-09
AIF1	0.41	0.00011
POSTN	0.44	7.20E-15

**Table 6:** DEGs in smooth muscle cell.

**Gene symbol**	**log2 fold change (cancer/normal)**	**adjusted *p*-value**
CFD	−1.42	0.0478
PTGDS	−1.37	0.0071
APOD	−1.01	0.0038
LUM	−1.00	0.0014
PTGS2	−0.86	1.73E-08
CCL4L2	−0.75	0.00035
SCGB1A1	−0.59	7.41E-36
RARRES1	−0.58	8.77E-15
VCAN	−0.49	6.95E-08
SERPINF1	−0.48	4.62E-05
MGST1	−0.45	4.40E-06
XCL2	−0.44	0.020
TNFAIP2	−0.43	1.22E-13
LEPR	−0.43	6.47E-08
TCIM	−0.41	0.0033
KRT17	−0.41	7.40E-05
ID1	−0.41	1.61E-07
KCNQ1OT1	−0.37	8.30E-14
SFRP1	−0.37	2.79E-05
TSHZ2	−0.35	5.06E-06
EFEMP1	−0.31	0.00099
TNFSF10	−0.31	3.93E-10
GEM	−0.31	0.013
NINJ1	−0.30	0.00023
TSC22D2	−0.29	0.0013
AC020916.1	−0.29	1.74E-09
TNXB	−0.29	2.02E-21
TSPYL2	−0.27	0.0024
IRS2	−0.27	0.00011
C1orf56	−0.27	2.61E-10
CXCL9	−0.27	3.91E-16
RGS16	−0.27	0.0067
GATA2	−0.27	0.00036
CST7	−0.26	7.85E-12
TRIB1	−0.26	8.24E-15
TGM2	−0.26	1.44E-07
CASQ2	0.31	9.06E-10

### Identification of formononetin’s targets for treating prostate cancer in single-cell level

**Figure 2:**
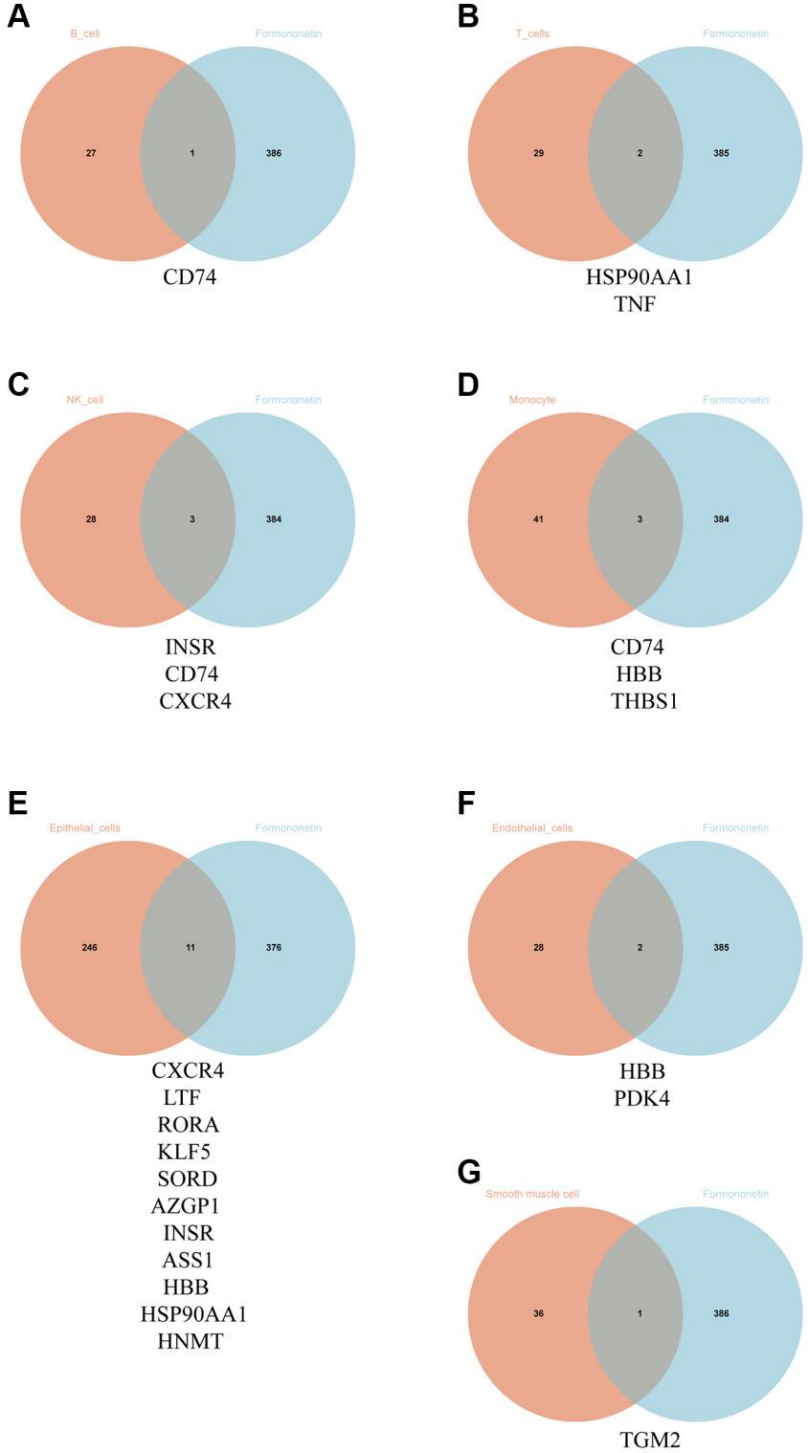
**Identification of formononetin’s targets against prostate cancer in single-cell level.** Venn diagram showed the number and gene symbol of common genes between formononetin and (**A**) B cell, (**B**) T cell, (**C**) NK cell, (**D**) monocyte, (**E**) epithelial cell, (**F**) endothelial cell, and (**G**) smooth muscle cell.

By searching the databases including Swiss Target Prediction database, PharmMapper database and SuperPred database, we identified 387 formononetin-associated genes after correction by using UniprotKB database. We then compared the formononetin-associated genes with the DEGs obtained from each cell type of prostate cancer. We found that formononetin could target DEGs in B cells included cluster of differentiation 74 (CD74) (Figure 2A); DEGs in T cell included heat shock protein 90 alpha family class A member 1 (HSP90AA1) and tumor necrosis factor (TNF) (Figure 2B); DEGs in NK cell included insulin receptor (INSR), CD74, and C-X-C motif chemokine receptor 4 (CXCR4) (Figure 2C); DEGs in monocyte included CD74, hemoglobin subunit beta (HBB), and thrombospondin 1 (THBS1) (Figure 2D); DEGs in epithelial cell included CXCR4, lactotransferrin (LTF), retinoic acid receptor-related orphan receptor alpha (RORA), Kruppel-like Factor 5 (KLF5), sorbitol dehydrogenase (SORD), alpha-2-glycoprotein 1, zinc-binding (AZGP1), INSR, argininosuccinate synthase 1 (ASS1), HBB, HSP90AA1, and histamine N-methyltransferase (HNMT) (Figure 2E); DEGs in endothelial cell included HBB and pyruvate dehydrogenase kinase 4 (PDK4) (Figure 2F); and DEGs in smooth muscle cell included and transglutaminase 2 (TGM2) (Figure 2G). Then, we grouped the immune cells including B cell, T cell, monocyte, and NK cell as cluster 1 (Table 7), giving 7 formononetin’s targets; and the tissue cells including epithelial cell, endothelial cell, and smooth muscle cell as cluster 2 (Table 7), giving 13 formononetin’s targets for further GO and KEGG analysis.

**Table 7:** Formononetin’s targets in immune cells and tissue cells.

**Immune cells**	**Tissue cells**
CD74	TGM2
HBB	HBB
THBS1	PDK4
INSR	CXCR4
CXCR4	LTF
HSP90AA1	RORA
TNF	KLF5
	SORD
	AZGP1
	INSR
	ASS1
	HSP90AA1
	HNMT

### Formononetin mediated immune response-related biological process and signaling pathways in the immune cell types of prostate cancer

**Figure 3:**
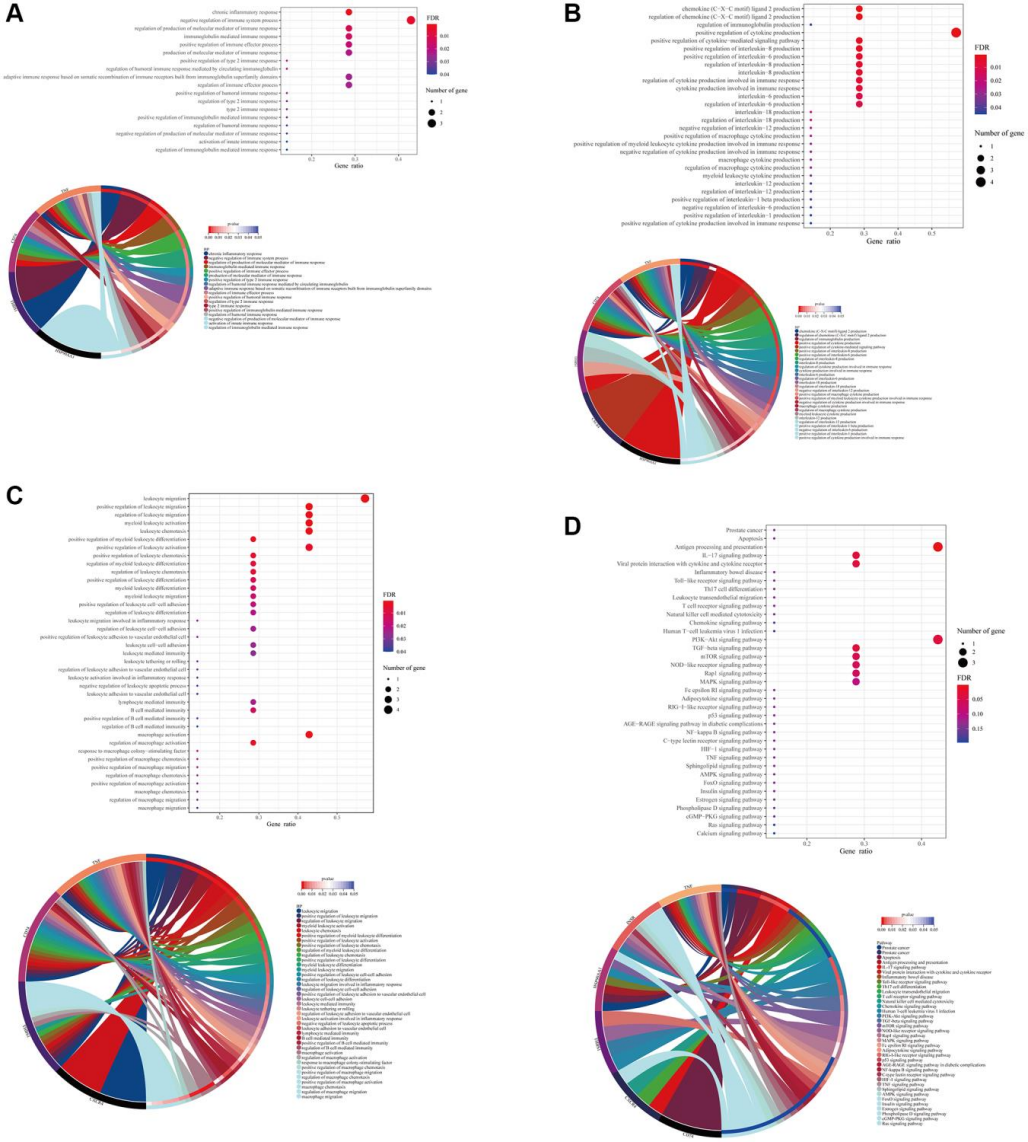
**Formononetin targeted genes related to immune responses in immune cells of prostate cancer.** Gene Ontology (GO) enrichment analysis showed the biological roles of formononetin in (**A**) immune response, (**B**) interleukins and cytokines production, and (**C**) leukocyte of immune cell cluster of prostate cancer. (**D**) Kyoto Encyclopedia of Genes and Genomes (KEGG) pathway enrichment analysis showed the roles of formononetin’s targets in apoptosis, and antigen presentation through the regulation of cell signaling pathways of immune cells. Lower panel of each figure was the Circos plot to show the involvement of gene in each item. The size of bubble represented the number of gene. The color of bubble represented the significance of the biological processes and pathways.

The result of GO analysis using the formononetin’s targets on the immune cell of prostate cancer highlighted the biological processes related to immune response (Figure 3A). It was regulated by different cytokines and interleukins production such as interleukin (IL)-1, IL-6, IL-12, and IL-18 (Figure 3B), leading to mediate the functions of different leukocytes such as lymphocyte mediated immunity, B cell mediated immunity, and macrophage activation (Figure 3C). KEGG pathway analysis further suggested the role of the formononetin’s targets in cell apoptosis and immune functions for treating prostate cancer (Figure 3D). All these functions were controlled by different cell signaling pathways such as PI3K-Akt signaling, TGF-beta signaling, mTOR signaling, and p53 signaling.

### Formononetin mediated metabolic process and signaling pathways in the tissue cell types of prostate cancer

**Figure 4:**
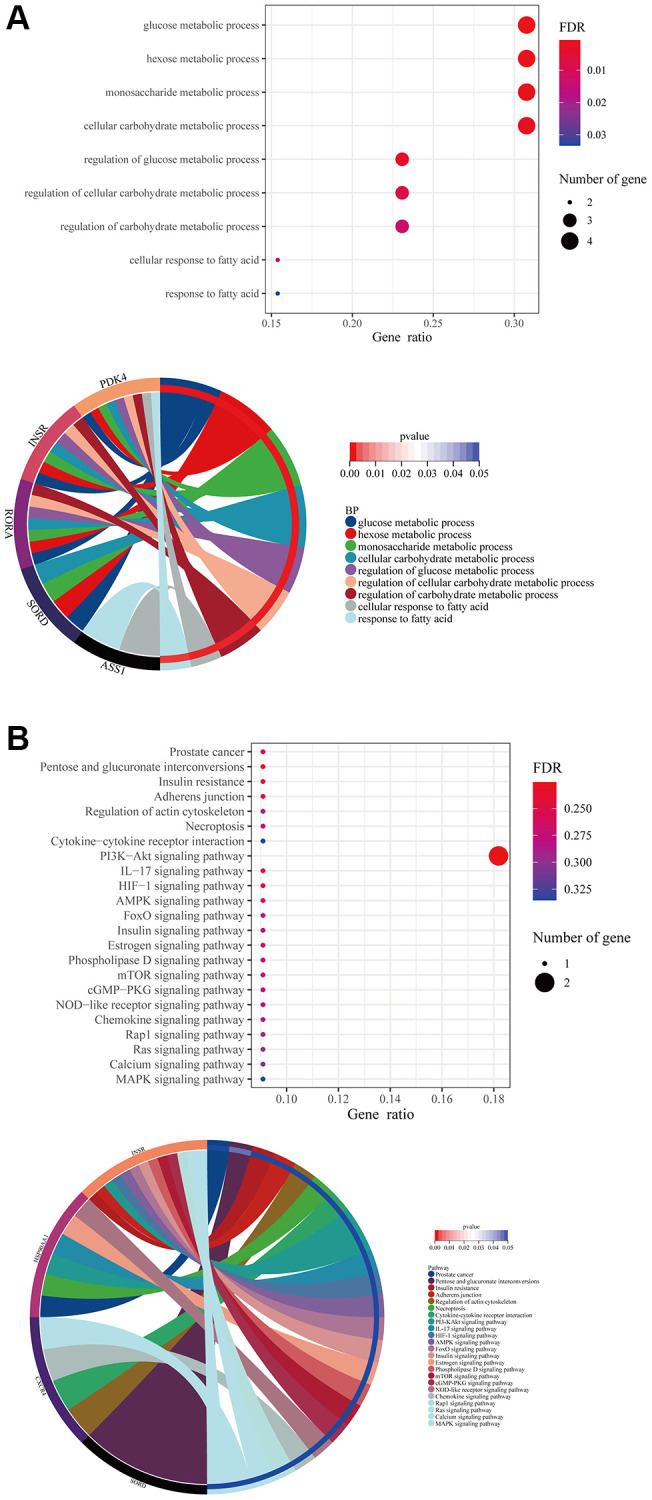
**Formononetin targeted genes related to metabolisms in tissue cells of prostate cancer.** (**A**) GO enrichment analysis showed the biological roles of formononetin in metabolisms of tissue cells cluster of prostate cancer. (**B**) KEGG enrichment analysis showed the roles of formononetin’s targets in insulin resistance, cell adhesion, and necroptosis through the regulation of cell signaling pathways of tissue cells. Lower panel of each figure was the Circos plot to show the involvement of gene in each item. The size of bubble represented the number of gene. The color of bubble represented the significance of the biological processes and pathways.

In the tissue cell, formononetin’s targets mainly contributed to the metabolisms such as glucose metabolic process, monosaccharide metabolic process, and cellular response to fatty acid (Figure 4A). Furthermore, the KEGG analysis highlighted the importance of formononetin’s targets in insulin resistance, adherens junction, cytokine-cytokine receptor interaction, and necroptosis via the regulation of cell signaling pathways (Figure 4B).

### Possible direct binding of formononetin with its target proteins CD74, INSR, TNF, and CXCR4

**Figure 5:**
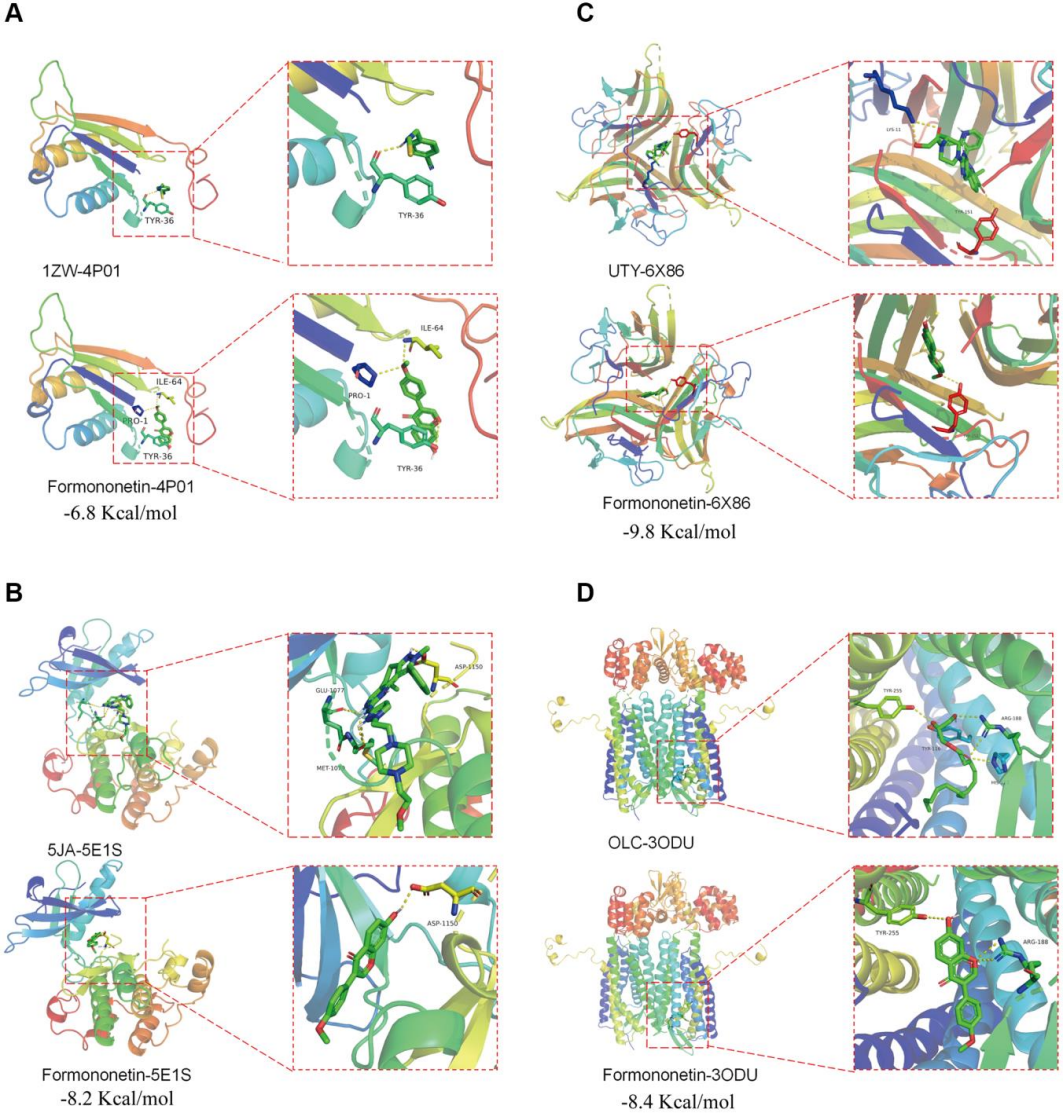
**Potential binding of formononetin with its target proteins TP53 and CDK1.** Molecular docking showed the binding of formononetin with (**A**) CD74 protein (PDB ID: 4P01), (**B**) INSR protein (PDB ID: 5E1S), (**C**) TNF protein (PDB ID: 6X86), and (**D**) CXCR4 protein (PDB ID: 3ODU).

The PDB database was searched for the protein structure of CD74, INSR, TNF, and CXCR4, and we found 4P01 [31], 5E1S [32], 6X86 [33], and 3OE0 [34] for docking analysis, respectively. An active cavity box model was established using PyMOL (version 2.5). For CD74 protein (PDB ID: 4P01), the active cavity box model was set at center x, y, z to −38.654, −14.151, 5.771, size x, y, z to 15.0, 15.0, 15.0, and the RMSD of the proto-ligand to 1.7 Å. We found that the formononetin could bind the CD74 protein (PDB ID: 4P01) through the formation of hydrogen bonds with the amino acid residues TYR-36 (3.5 Å), ILE-64 (3.2 Å), PRO-1 (3.2 Å) and its free docking energy with the protein was −6.8 Kcal/mol (Figure 5A). For INSR protein (PDB ID: 5E1S), the active cavity box model was set to center x, y, z of 3.538, 19.827, 21.984, size x, y, z of 15.0, 15.0, 15.0, and RMSD of the proto-ligand of 2.1 Å. Formononetin formed a hydrogen bond with amino acid residue ASP-1150 (2.2 Å) of INSR protein (PDB ID: 5E1S), and its free docking energy with the protein was −8.2 Kcal/mol (Figure 5B). For TNF protein (PDB ID: 6X86), the active cavity box model was set with center x, y, z of 55.474, −6.73, 9.924, size x, y, z of 15.0, 15.0, 15.0, the RMSD of the proto-ligand was only one and calculated to be 0.000 Å. Formononetin formed hydrogen bond with amino acid residue TYR-151 (2.9 Å) of TNF protein (PDB ID:6X86) and its free docking energy with the protein was −9.8 Kcal/mol (Figure 5C). For CXCR4 protein (PDB ID: 3ODU), the active cavity box model was set with center x, y, z of −13.203, 15.376, 71.731, size x, y, z of 15.0, 15.0, 18.75, and RMSD of the proto-ligand of 2.1 Å. Formononetin formed hydrogen bond with amino acid residue TYR-225 (3.4 Å), ARG-188 (3.2 Å) of CXCR4 protein (PDB ID: 3ODU) and its free docking energy with the protein was −8.4 Kcal/mol (Figure 5D). Taken together, our results suggested a possible direct binding of formononetin with its target proteins CD74, INSR, TNF, and CXCR4.

### Validation of network pharmacology’s result

**Figure 6:**
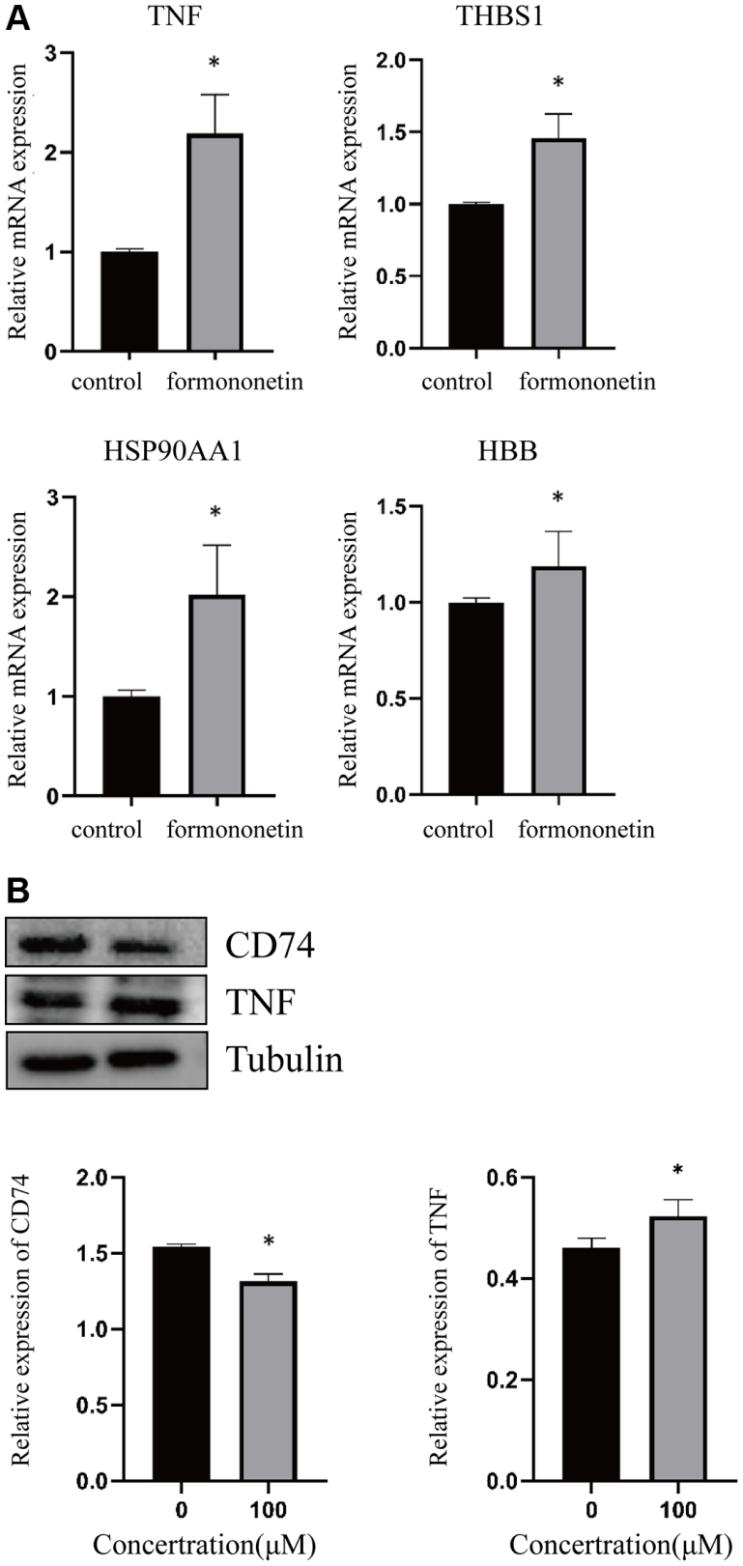
**Formononetin altered the expression of its targets in prostate cancer cell line.** (**A**) qPCR analysis showed that formononetin induced the expression of mRNA of TNF, THBS1, HSP90AA1, and HBB in prostate cancer cell. (**B**) Formononetin treatment also induced the protein level of TNF and reduced the protein level of CD74 in prostate cancer cell.

In order to validate the finding of network pharmacology, we treated the human prostate cancer cell DU145 with formononetin, followed by qPCR analysis and WB. The result of qPCR analysis showed that the treatment of formononetin could induce the mRNA expression of TNF, THBS1, HSP90AA1, and HBB (Figure 6A). To further validate the effect of formononetin on the stability of its target proteins, Western blotting was performed. The result showed that treatment of formononetin could increase the protein level of TNF, but decrease the protein level of CD74 (Figure 6B), suggesting the binding of formononetin may regulate the stability of its target proteins.

## DISCUSSION

Formononetin, a natural flavonoid with antioxidant and cellular therapeutic properties, is a potential drug for treating prostate cancer. *In vitro* studies demonstrated that formononetin inhibited the proliferation and induced apoptosis of prostate cancer cell PC-3 through the induction of Bax/Bcl-2 ratio and regulation of p38/Akt pathway [35]. Also, formononetin was reported to induce the early apoptosis of prostate cancer cell DU145 via the regulation of mitochondrial apoptotic pathway and downregulation of IGF-1/IGF-1R signaling pathway [16, 36]. The results of a clinical intervention study carried out by Jarred et al. showed that dietary isoflavones supplement formononetin increased apoptosis in low and intermediate prostate cancer with minimal adverse effects [37]. In addition, Dong et al. found that the combined use of docetaxel and formononetin nanoparticles could effectively reduce side effects during the prostate cancer treatment [38]. So, formononetin should be a promising drug with potential anti-prostate cancer properties.

In order to understand the detail anti-prostate cancer effect of formononetin in single-cell level, we combined and analyzed the single cell sequencing data of prostate cancer obtained from 2 studies [19, 20]. When we compared the expression profile between tumoral and adjacent normal tissue in single-cell level, we identified the DEGs in 2 cell clusters, including immune cells (B cell, T cell, monocyte, and NK cell) and tissue cell (endothelial cell, epithelial cell, and smooth muscle cell). The identification of these DEGs could be served as prognostic marker in prostate cancer. Indeed, some of the identified genes were reported to be associated with other cancer types, but not prostate cancer. For instance, we found the overexpression of immunoglobulin kappa C (IGKC) in NK cell (Table 2). A study of non-small cell lung cancer patient showed the overexpression of IGKC in stroma-infiltrating plasma cells [39]. Another study conducted by Schmidt et al. evaluating the expression of IGKC in 909 early breast cancer demonstrated its association with distant disease-free survival of patients [40]. Similar findings were observed in node-negative breast cancer that the overexpression of IGKC was significantly associated with DFS, especially in ER negative and in luminal B carcinomas [41]. Our data suggested that IGKC could be a potential biomarker for prognosis of prostate cancer.

In the later part of study, we aimed to determine the possible use of formononetin for treating prostate cancer in single-cell level. By using the network pharmacology, we found the formononetin could target different cell types specifically. In the immune cell cluster of prostate cancer, formononetin targeted CD74, TNF, HBB, THBS1, INSR, CXCR4, and HSP90AA1. Some of them were reported to play important roles in tumorigenesis. Our result showed that formononetin could target and reduce the expression of CD74, which is a cell surface membrane receptor of cytokine macrophage migration inhibitory factor (MIF) [42]. CD74 play multiple roles in the immune system such as antigen presentation and B cell differentiation [43], and cumulating studies demonstrated the roles of CD74 in tumorigenesis. Xu et al. showed the association of CD74 with malignancies and immune microenvironment in gliomas [44]. A study of human breast cancer showed that CD74 interacted with CD44 to promote tumorigenesis and metastasis of MDA-MB-231 cell through the regulation of RHOA [45]. In addition, overexpressed CD74 was reported to interplay with MIF to promote the tumor growth in advanced melanoma patients, and its elevated expression was associated with the poorer patient survival [46]. A similar result was observed in the study of hepatocellular carcinoma (HCC) conducted by Xiao et al. that stromal CD74+ macrophages enrichment was associated with favorable prognosis in patients with HCC [47]. CD74 was found to be overexpressed in prostate cancer cell DU145, as compared with normal prostate cells, and blocking interaction of MIF with CD74 selectively inactivated ERK1/2, leading to reduced prostate cancer cell proliferation and increased apoptosis [48]. On the other hand, overexpression of the receptor CD74 was closely associated with growth and migration of prostate cancer cells [49]. These studies suggested that formononetin targeting CD74 could be effective strategies for prostate cancer therapy.

In addition, our result showed that formononetin could target and induce the expression of TNF in prostate cancer cell. TNF is an inflammatory cytokine that play dual roles in cancer, however its role in prostate cancer is still largely unknown. But TNF was reported to be cytotoxic to tumour cells and destroy tumour blood vessels [50]. In addition, the combined effect of TNF and ionising radiation on the induction of apoptosis in bladder cancer cells was demonstrated [51]. On the other hands, we found that formononetin could target THBS1 and HBB. THBS1, an adhesive glycoprotein, played roles in anti-angiogenesis and anti-tumorigenesis [52]. In prostate cancer, THBS1 inhibited neovascularization and tumor growth [53]. Also, THBS1 played a key role in the regulation of prostate epithelial and stromal growth by inhibiting angiogenesis and activating latent TGF-beta [54]. Jin et al. demonstrated that overexpression of THBS1 inhibited the growth of DU145 tumors in Balb/c mice [55]. Vice versa, the knockdown of THBS1 increased the growth and colony forming ability of prostate cancer cell [56]. HBB is one of the components of the bead protein chain of haemoglobin A, and its basic function is oxygen transport. A study on gene expression profile of anaplastic thyroid cancer cell lines (ACL) showed significant reduced expression of HBB in ACL [57]. Functionally, overexpression of HBB could suppress the growth of ACL. A similar result was observed that induced HBB expression inhibited growth and metastasis of neuroblastoma [58], suggesting the anti-tumor roles of HBB.

In our analysis, we also predicted the formononetin’s targets such as INSR and AZGP1 to control the metabolisms in the tissue cell of prostate cancer. And they were reported to play roles in tumorigenesis. For instance, a study using a genomic screen of the tumour vasculature showed the involvement of INSR in tumour angiogenesis [59]. INSR was found to be overexpressed in angiogenic vasculature of human tumors and the was correlated to shorter survival of cancer patient. In prostate cancer, the induced expression of INSR could increase cell proliferation, colony formation, migration, invasion and resistance to apoptosis in prostate cancer cells through the cooperation with IGF1R [60]. A similar finding from Ofer’s group demonstrated that knockdown of INSR reduced cell growth and proliferation of prostate cancer cell, as well as driving cells into apoptosis [61]. Other than INSR, our result highlighted that AZGP1 was targeted by formononetin and played major role in lipid metabolism. A tissue microarray containing 11,152 prostate cancers showed that the reduced AZGP1 expression was associated with adverse prostate cancer prognosis through the regulation of PTEN [62]. A similar result was obtained from a Chip-Seq study that AZGP1 acted as an androgen-responsive gene to mediate proliferation and metastasis of prostate cancer cell via the contribution of androgen receptor [63]. In addition, a clinical study included 191 patients who underwent androgen deprivation therapy showed that low AZGP1 expression was associated with a shorter survival time in prostate cancer patients [64]. Also, the low expression of AZGP1 could be used to predict the recurrence of margin-positive, localized prostate cancer [65].

For the limitation of the study, our results were mainly obtained from the *in silico* analysis. Although the findings were validated by using prostate cancer cell line, the involvement of formononetin’s targets in prostate cancer in single-cell level was still difficult to confirm. In addition, there were many challenges in translating these findings to clinical applications. For instance, the single use of formononetin may be not effective to treat the cancer. So, future study could focus on the combined therapy using formononetin. In addition, further clinical trials exploring the use of formononetin in prostate cancer are essential. Moreover, further biochemistry experiments should be carried out to confirm the binding of formononetin to its target proteins. In conclusion, for the first time, our result suggested that formononetin could target the candidates involved in the tumorigenesis of prostate cancer in single-cell level. It provided a ground information for further study on formononetin for treating prostate cancer.

## Supplementary Materials

Supplementary Table 1
