# Supplementary material for: Therapeutic targets of formononetin for treating prostate cancer at the single-cell level
Source: Aging (Albany NY). 2024 Jun 13;16(12):10380–401. doi: 10.18632/aging.205935 (PMC11236323; doi:10.18632/aging.205935)
Supplement: Supplementary Table 1 [file aging-16-205935-s001.docx]

**Supplementary Table 1. DEGs in epithelial cell.**

| Gene symbol | log2 fold change (cancer/normal) | adjusted p-value |
| --- | --- | --- |
| SCGB1A1 | -2.96 | 2.66E-46 |
| OLFM4 | -1.16 | 7.50E-13 |
| SCGB3A1 | -1.09 | 1.03E-51 |
| PPP1R15A | -0.91 | 5.91E-10 |
| LCN2 | -0.84 | 3.20E-24 |
| KLF6 | -0.84 | 6.95E-13 |
| LTF | -0.83 | 1.08E-76 |
| BTG2 | -0.83 | 6.00E-27 |
| TFF1 | -0.81 | 2.27E-45 |
| S100P | -0.80 | 8.98E-33 |
| DUSP4 | -0.79 | 7.19E-14 |
| FOSB | -0.77 | 3.45E-21 |
| GDF15 | -0.77 | 3.01E-08 |
| GADD45B | -0.74 | 4.78E-15 |
| ZFP36L2 | -0.74 | 4.00E-07 |
| REL | -0.72 | 2.55E-18 |
| JUN | -0.71 | 2.04E-32 |
| NR4A2 | -0.69 | 4.45E-17 |
| AQP3 | -0.68 | 7.36E-07 |
| DNAJB1 | -0.68 | 1.56E-13 |
| CTSH | -0.67 | 1.71E-12 |
| DUSP2 | -0.66 | 9.08E-33 |
| INTS6 | -0.66 | 1.25E-34 |
| ASS1 | -0.66 | 5.45E-15 |
| MTRNR2L8 | -0.65 | 4.13E-33 |
| CD55 | -0.65 | 5.82E-11 |
| C19orf33 | -0.65 | 3.41E-36 |
| GPRC5A | -0.65 | 8.65E-21 |
| KRT23 | -0.64 | 1.82E-09 |
| PIGR | -0.64 | 1.48E-07 |
| TM4SF1 | -0.63 | 4.01E-13 |
| JUNB | -0.62 | 1.09E-32 |
| IGFBP3 | -0.62 | 2.36E-49 |
| IER5 | -0.62 | 6.84E-42 |
| NFKBIA | -0.61 | 1.37E-11 |
| CYP4B1 | -0.61 | 3.08E-82 |
| CD82 | -0.60 | 8.59E-10 |
| CD81 | -0.60 | 0.0073 |
| TNFAIP2 | -0.60 | 5.78E-35 |
| SDC4 | -0.60 | 4.62E-41 |
| DUSP5 | -0.59 | 3.70E-27 |
| NCOA7 | -0.59 | 1.11E-22 |
| MGP | -0.58 | 0.0063 |
| ETS2 | -0.58 | 1.31E-35 |
| S100A6 | -0.58 | 8.36E-17 |
| CD69 | -0.56 | 2.11E-112 |
| RHOV | -0.56 | 5.37E-60 |
| DDIT4 | -0.55 | 5.03E-74 |
| IRF1 | -0.55 | 2.93E-07 |
| SDC1 | -0.55 | 2.93E-09 |
| KRT13 | -0.53 | 1.46E-32 |
| ZFP36L1 | -0.53 | 2.60E-05 |
| MAFF | -0.53 | 1.13E-20 |
| FAM3D | -0.52 | 1.40E-66 |
| CRABP2 | -0.52 | 1.58E-49 |
| MEIS2 | -0.51 | 2.48E-24 |
| IER2 | -0.51 | 1.56E-10 |
| APP | -0.51 | 3.18E-05 |
| KRT7 | -0.50 | 0.00024 |
| SOCS3 | -0.50 | 0.021 |
| MMP7 | -0.50 | 1.02E-08 |
| SERPINB1 | -0.50 | 0.00027 |
| HSP90AA1 | -0.49 | 1.80E-07 |
| CXCR4 | -0.48 | 9.57E-82 |
| CMYA5 | -0.47 | 5.39E-13 |
| PLPP2 | -0.47 | 1.11E-17 |
| S100A16 | -0.47 | 0.0031 |
| CLDN7 | -0.47 | 6.54E-15 |
| KLF5 | -0.47 | 8.88E-40 |
| TSPYL2 | -0.47 | 2.34E-45 |
| NRARP | -0.46 | 9.12E-41 |
| C1orf56 | -0.46 | 2.23E-65 |
| CFD | -0.46 | 4.74E-29 |
| FOS | -0.46 | 1.37E-27 |
| ARL4C | -0.46 | 3.50E-111 |
| UGCG | -0.46 | 2.23E-28 |
| TFCP2L1 | -0.46 | 3.63E-37 |
| CLU | -0.46 | 0.021 |
| MACC1 | -0.45 | 2.89E-40 |
| ACTB | -0.45 | 2.62E-21 |
| PLSCR1 | -0.45 | 0.00058 |
| DUSP1 | -0.45 | 0.0013 |
| PLAT | -0.45 | 4.93E-28 |
| ZFP36 | -0.44 | 9.22E-10 |
| KDM6B | -0.44 | 3.80E-30 |
| AC020916.1 | -0.44 | 5.91E-08 |
| EPHX1 | -0.43 | 2.03E-35 |
| CCND1 | -0.43 | 1.20E-30 |
| PSCA | -0.43 | 2.68E-94 |
| CTSS | -0.42 | 6.63E-06 |
| TCIM | -0.42 | 6.92E-53 |
| LIMA1 | -0.42 | 2.06E-25 |
| TNFSF15 | -0.42 | 9.33E-31 |
| B4GALT1 | -0.42 | 5.57E-18 |
| SELENBP1 | -0.42 | 1.49E-34 |
| TOB1 | -0.41 | 3.05E-17 |
| FOXQ1 | -0.41 | 4.57E-56 |
| LAMB3 | -0.41 | 1.10E-08 |
| MPZL2 | -0.40 | 5.64E-44 |
| MDK | -0.40 | 6.81E-15 |
| CP | -0.40 | 2.41E-40 |
| HSD17B13 | -0.40 | 1.06E-99 |
| WEE1 | -0.40 | 8.37E-63 |
| S100A4 | -0.39 | 2.16E-81 |
| RND3 | -0.39 | 1.11E-25 |
| KLF2 | -0.39 | 2.19E-78 |
| S100A14 | -0.39 | 5.45E-22 |
| TRAC | -0.39 | 1.14E-127 |
| PPP1R1B | -0.39 | 1.25E-37 |
| ID3 | -0.38 | 0.0090 |
| CXADR | -0.38 | 2.34E-29 |
| ELF3 | -0.38 | 6.45E-11 |
| MYOF | -0.38 | 2.20E-33 |
| AC007906.2 | -0.37 | 5.39E-34 |
| TRIM29 | -0.37 | 0.00097 |
| IL32 | -0.37 | 8.13E-23 |
| CRIM1 | -0.37 | 4.54E-33 |
| GABRE | -0.37 | 1.32E-66 |
| ATP2B1 | -0.37 | 0.0038 |
| EMP1 | -0.36 | 7.73E-22 |
| PDLIM1 | -0.36 | 0.00017 |
| F3 | -0.36 | 2.02E-28 |
| LGALS3BP | -0.36 | 1.65E-16 |
| CFH | -0.35 | 2.36E-17 |
| KRT19 | -0.35 | 3.24E-11 |
| TUBA4A | -0.35 | 3.05E-22 |
| VSIG2 | -0.35 | 1.15E-83 |
| BCL11A | -0.35 | 0.020 |
| IFITM3 | -0.35 | 9.83E-06 |
| TKT | -0.34 | 6.35E-11 |
| SOX9 | -0.34 | 8.28E-33 |
| ARRDC3 | -0.34 | 0.0038 |
| CDKN1A | -0.34 | 2.44E-08 |
| SMIM22 | -0.34 | 5.29E-34 |
| GLUL | -0.34 | 3.28E-11 |
| LMNA | -0.34 | 1.43E-12 |
| HLA-DMA | -0.33 | 3.97E-18 |
| IL18 | -0.33 | 4.64E-61 |
| CD59 | -0.33 | 0.0050 |
| PDE4D | -0.33 | 1.60E-09 |
| INSR | -0.33 | 2.76E-19 |
| KCNQ1OT1 | -0.33 | 5.76E-19 |
| TNFRSF12A | -0.33 | 9.49E-10 |
| CCL5 | -0.33 | 1.80E-49 |
| MID1IP1 | -0.33 | 3.42E-28 |
| LMO4 | -0.32 | 8.27E-23 |
| RDH10 | -0.32 | 2.12E-27 |
| DMKN | -0.32 | 0.00022 |
| CLDN3 | -0.32 | 6.99E-06 |
| DSP | -0.32 | 3.58E-09 |
| S100A9 | -0.32 | 9.75E-10 |
| GBP2 | -0.32 | 5.20E-07 |
| FCGRT | -0.32 | 0.0024 |
| BIRC3 | -0.31 | 1.33E-49 |
| ITGA2 | -0.31 | 1.62E-26 |
| PYCARD | -0.31 | 9.67E-14 |
| GMNN | -0.31 | 5.55E-26 |
| TUBA1A | -0.31 | 1.76E-32 |
| LTB | -0.31 | 9.99E-132 |
| ENAH | -0.31 | 1.38E-19 |
| GPRC5C | -0.31 | 2.45E-13 |
| PLK2 | -0.31 | 2.59E-13 |
| HPGD | -0.31 | 1.21E-06 |
| PHLDA2 | -0.31 | 8.97E-44 |
| PDZK1IP1 | -0.30 | 5.44E-69 |
| S100A2 | -0.30 | 2.41E-11 |
| CEACAM1 | -0.30 | 2.11E-17 |
| HBEGF | -0.30 | 5.67E-67 |
| NINJ1 | -0.30 | 2.09E-07 |
| HIST1H4C | -0.30 | 9.54E-09 |
| TIMP1 | -0.29 | 0.00020 |
| SCUBE2 | -0.29 | 9.25E-08 |
| CX3CL1 | -0.29 | 5.36E-14 |
| TENT5A | -0.29 | 1.56E-61 |
| CAV2 | -0.29 | 2.63E-63 |
| IER5L | -0.29 | 2.11E-34 |
| C1orf21 | -0.29 | 0.017 |
| MX1 | -0.29 | 2.82E-07 |
| TSPAN13 | -0.28 | 6.85E-32 |
| TSC22D2 | -0.28 | 7.83E-13 |
| SPARCL1 | -0.28 | 0.012 |
| RORA | -0.28 | 4.00E-56 |
| TRBC2 | -0.28 | 9.54E-95 |
| MARCKS | -0.28 | 0.0030 |
| MGST2 | -0.28 | 9.54E-07 |
| GAS6 | -0.28 | 1.21E-07 |
| DSC3 | -0.28 | 4.03E-08 |
| EPCAM | -0.28 | 4.99E-15 |
| SNCG | -0.27 | 1.35E-14 |
| NET1 | -0.27 | 3.47E-20 |
| MECOM | -0.27 | 1.29E-11 |
| HNMT | -0.27 | 0.0017 |
| CYTOR | -0.27 | 2.40E-87 |
| SLC14A1 | -0.27 | 0.038 |
| CREB3L1 | -0.27 | 3.97E-18 |
| EHD4 | -0.27 | 0.0015 |
| LXN | -0.27 | 1.13E-29 |
| CXCL1 | -0.27 | 6.79E-54 |
| CEMIP2 | -0.26 | 2.11E-39 |
| SRGN | -0.26 | 5.71E-123 |
| ZC3H12A | -0.26 | 5.06E-06 |
| PBX1 | -0.26 | 1.05E-13 |
| OVOL1 | -0.26 | 4.15E-80 |
| TFRC | -0.26 | 0.00019 |
| ETV3 | -0.26 | 3.24E-18 |
| AC058791.1 | -0.26 | 4.62E-30 |
| TRBC1 | -0.25 | 4.25E-92 |
| HIST1H2AC | 0.26 | 5.50E-05 |
| TMSB15A | 0.26 | 2.24E-22 |
| VEGFA | 0.28 | 6.18E-33 |
| AC020571.1 | 0.29 | 0.00063 |
| TRPM8 | 0.30 | 1.42E-05 |
| ZNF532 | 0.30 | 0.00076 |
| CALD1 | 0.30 | 0.0014 |
| ALOX15B | 0.32 | 1.45E-05 |
| APOE | 0.32 | 7.79E-82 |
| LINC00844 | 0.33 | 1.22E-20 |
| CRYAB | 0.33 | 1.48E-05 |
| CYB5A | 0.35 | 2.75E-31 |
| NME4 | 0.35 | 6.38E-05 |
| BASP1 | 0.35 | 5.31E-14 |
| IDH1 | 0.35 | 4.26E-05 |
| H2AFJ | 0.37 | 4.14E-24 |
| MESP1 | 0.40 | 2.86E-28 |
| AZGP1 | 0.40 | 2.52E-22 |
| RAB3B | 0.42 | 8.78E-12 |
| ARG2 | 0.42 | 4.59E-11 |
| PLPP1 | 0.42 | 9.56E-11 |
| RDH11 | 0.44 | 1.12E-07 |
| SMS | 0.45 | 1.55E-12 |
| SEC11C | 0.45 | 0.00037 |
| CPE | 0.47 | 2.19E-16 |
| STEAP2 | 0.48 | 1.26E-30 |
| CKB | 0.49 | 1.68E-29 |
| HBA1 | 0.50 | 1.21E-06 |
| TMPRSS2 | 0.52 | 8.75E-31 |
| PLA2G2A | 0.53 | 1.62E-20 |
| NKX3-1 | 0.53 | 1.60E-29 |
| TFF3 | 0.53 | 6.25E-40 |
| MT2A | 0.54 | 1.03E-21 |
| HBA2 | 0.56 | 1.40E-10 |
| DBI | 0.60 | 6.25E-40 |
| NDRG1 | 0.61 | 6.75E-14 |
| IGLC2 | 0.63 | 4.13E-39 |
| LINC01297 | 0.67 | 1.62E-48 |
| PMEPA1 | 0.68 | 2.27E-23 |
| NEFH | 0.75 | 1.12E-37 |
| SLC45A3 | 0.77 | 3.93E-05 |
| MT1E | 0.80 | 4.31E-08 |
| KLK4 | 0.89 | 2.54E-26 |
| MSMB | 0.90 | 2.36E-58 |
| SORD | 0.93 | 6.29E-26 |
| HBB | 0.94 | 2.77E-14 |
| KLK2 | 1.07 | 9.21E-60 |
| KLK3 | 1.10 | 6.70E-58 |
| ACPP | 1.15 | 3.73E-55 |
| MT1G | 1.41 | 3.58E-43 |
